# Supplementary material for: Structural Analysis of the Drosophila melanogaster GSTome
Source: Biomolecules. 2024 Jun 26;14(7):759. doi: 10.3390/biom14070759 (PMC11274691; doi:10.3390/biom14070759)
Supplement: Supplementary file 1 [file biomolecules-14-00759-s001.zip › biomolecules-3066182-supplementary.pdf]

Article

Supplementary Materials

Structural Analysis of the Drosophila Melanogaster GSTome

Nicolas Petiot[1], Mathieu Schwartz [2], Patrice Delarue[1], Patrick Senet[1], Fabrice Neiers [2] and Adrien Nicolaï [1,*]

[1] Laboratoire Interdisciplinaire Carnot de Bourgogne, UMR 6303 CNRS-Université de Bourgogne, 21078 Dijon, France
[2] Centre des Sciences du Goût et de l'Alimentation, Université de Bourgogne, INRA, CNRS, 21000 Dijon, France
* adrien.nicolai@u-bourgogne.fr

Citation: Title. *Journal Not Specified* **2024**, *1*, 0. https://doi.org/

Received:
Revised:
Accepted:
Published:

Copyright: © 2024 by the authors. Submitted to *Journal Not Specified* for possible open access publication under the terms and conditions of the Creative Commons Attribution (CC BY) license (https://creativecommons.org/licenses/by/4.0/).

List of Tables

S1. Summary of GSH ligand structures extracted from AlphaFill predictions. . . 2

List of Figures

S1. Multiple Sequence Alignment of the complete *D. mel* GSTome. 3
S2. Position of positively (yellow) and negatively (purple) charged amino acids as a function of MSA number. 4
S3. Cartoon representation of GST structures from D. mel and measured by X-ray crystallography. 5
S4. Cartoon representation of GST structures of class δ from *D. mel* and predicted by AlphaFold. 6
S5. Cartoon representation of GST structures of class ε from *D. mel* and predicted by AlphaFold. 7
S6. Cartoon representation of GST structures of class ω from *D. mel* and predicted by AlphaFold. 8
S7. Cartoon representation of GST structures of class σ from *D. mel* and predicted by AlphaFold. 9
S8. Cartoon representation of GST structures of class θ from *D. mel* and predicted by AlphaFold. 10
S9. Cartoon representation of GST structures of class ζ from *D. mel* and predicted by AlphaFold. 11
S10. Radius of gyration (in Å) as a function of GST class for the D. mel GSTome . 12
S11. Charge analysis and conservation plot of the residues in Dimerization interface (orange) and in the Glutathione Binding Site (purple). 13
S12. Thermal B-factors (in Å^2) and their standard deviation (in Å^2) as a function of MSA number computed for the 12 GST experimental structures presented in Tab. 1. 14

Table S1. Summary of GSH ligand structures extracted from AlphaFill predictions.

Protein	Uniprot code	Identity query	Number of GSH
GstD1	P20432	50%	9
GstD2	Q9VG98	50%	9
GstD3	Q9VG97	50%	3
GstD4	Q9VG96	50%	9
GstD5	Q9VG95	50%	9
GstD6	Q9VG94	40%	10
GstD7	Q9VG93	50%	3
GstD8	Q9VG92	50%	9
GstD9	Q9VGA0	50%	5
GstD10	Q9VGA1	50%	9
GstD11iA	Q8SXQ9	40%	9
GstE1	Q7KK90	40%	11
GstE2	Q7JYZ9	50%	3
GstE3	A1ZB68	50%	5
GstE4	A1ZB69	50%	3
GstE5	A1ZB70	50%	3
GstE6	A1ZB71	50%	3
GstE7	A1ZB72	40%	6
GstE8	A1ZB73	40%	3
GstE9	Q7K8X7	50%	3
GstE10	Q4V6J1	40%	3
GstE11	Q7JVZ8	40%	10
GstE12	Q9XYZ9	50%	5
GstE13	Q7JVI6	30%	29
GstE14	Q7JYX0	50%	2
GstO1	Q9VSL6	30%	5
GstO2iA	Q9VSL5	30%	10
GstO2iB	Q9VSL4	30%	1
GstO3	Q9VSL2	30%	5
GstS1	P41043	40%	2
GstT1	Q7K0B6	30%	5
GstT2	A1Z7X7	30%	3
GstT3	E1JJS1	25%	1
GstT4	Q8MRM0	25%	2
GstZ1	Q9VHD3	40%	3
GstZ2	Q9VHD2	40%	4

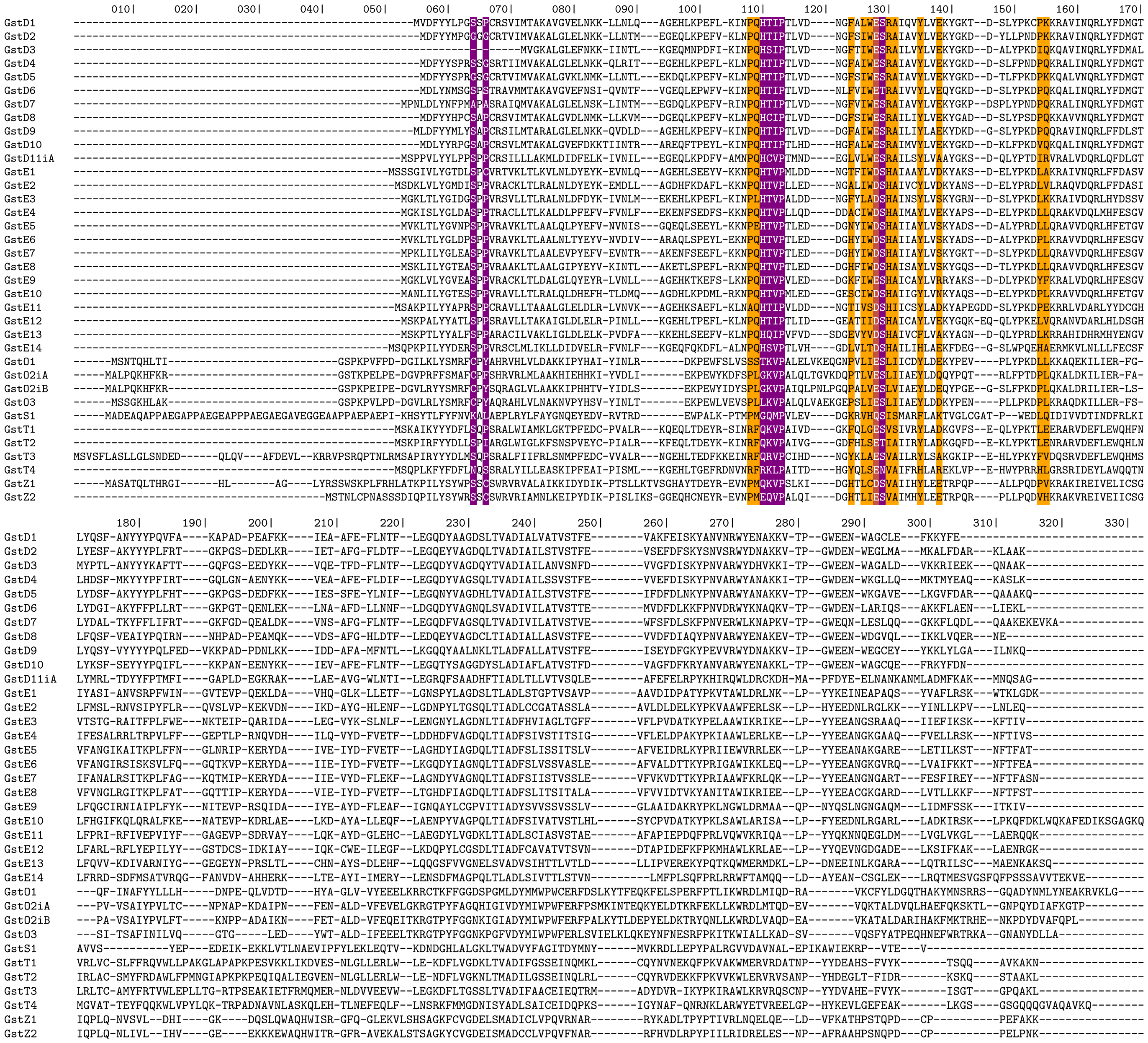

Figure S1. Multiple Sequence Alignment of the complete *D. mel* GSTome.

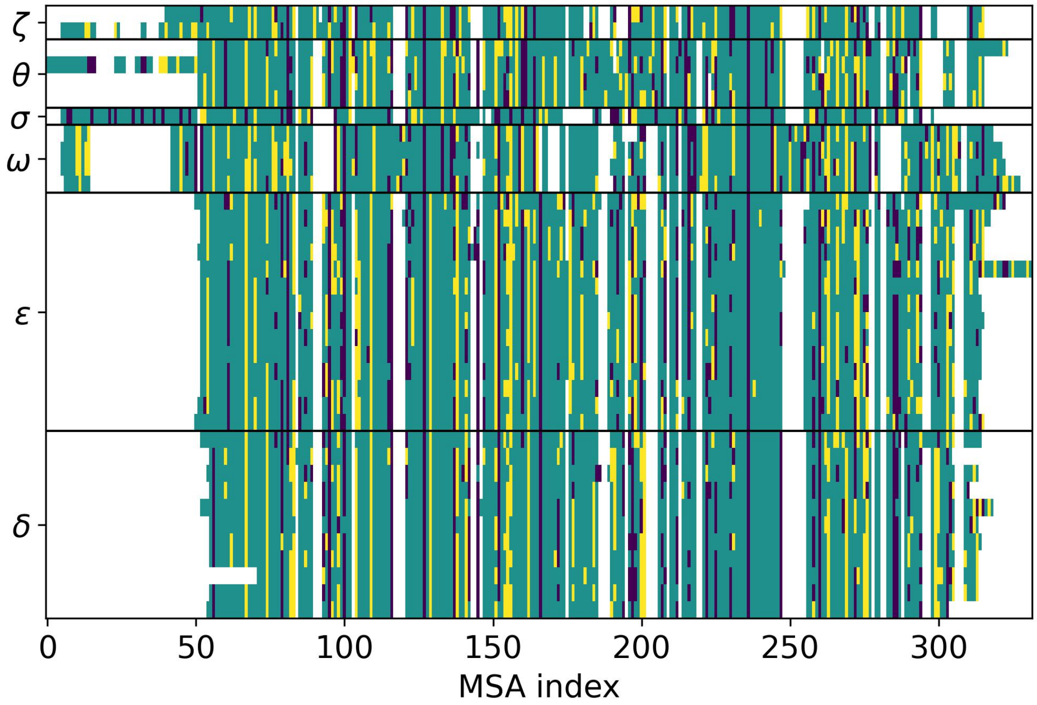

Figure S2. Position of positively (yellow) and negatively (purple) charged amino acids as a function of MSA number.

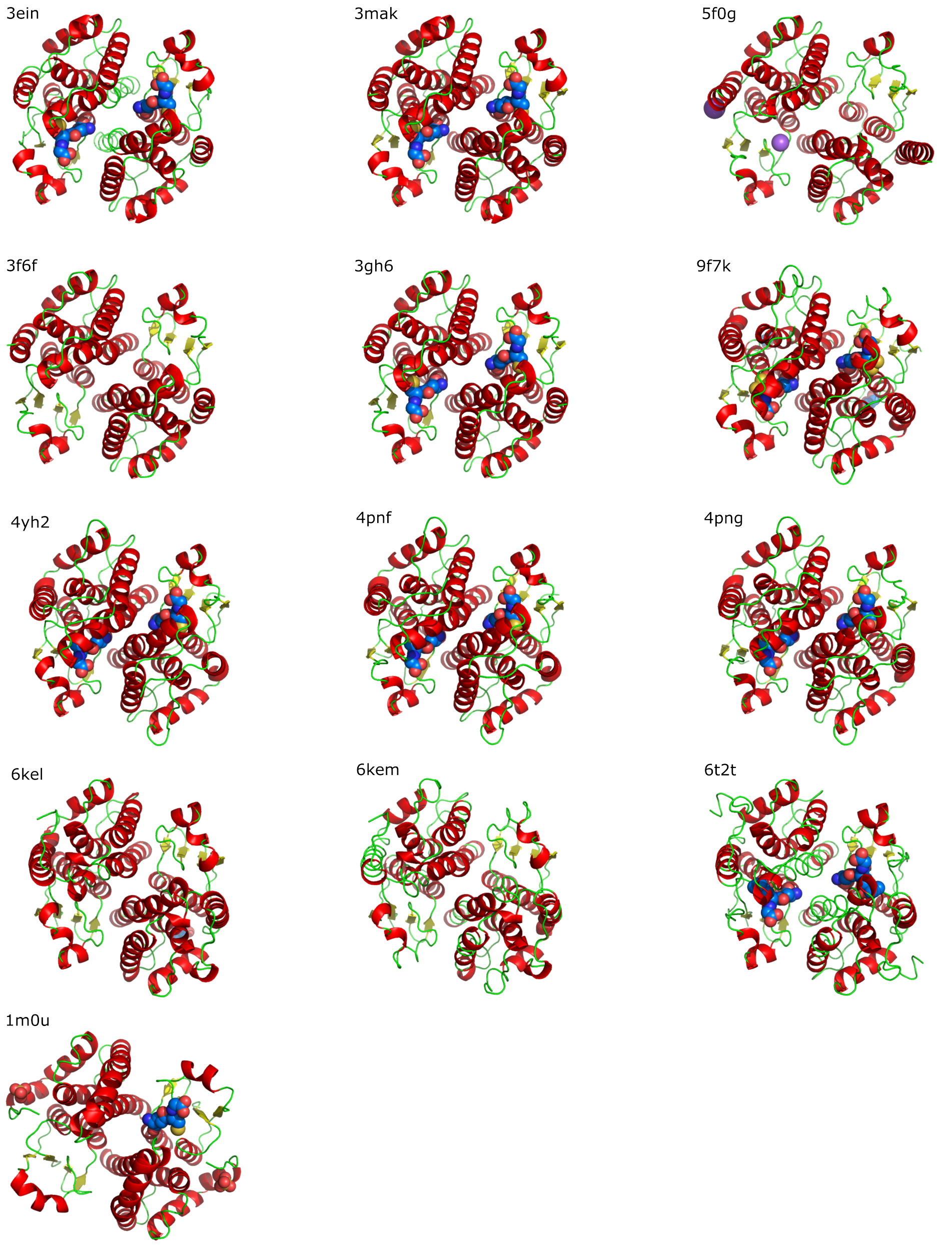

Figure S3. Cartoon representation of GST structures from D. mel and measured by X-ray crystallography.

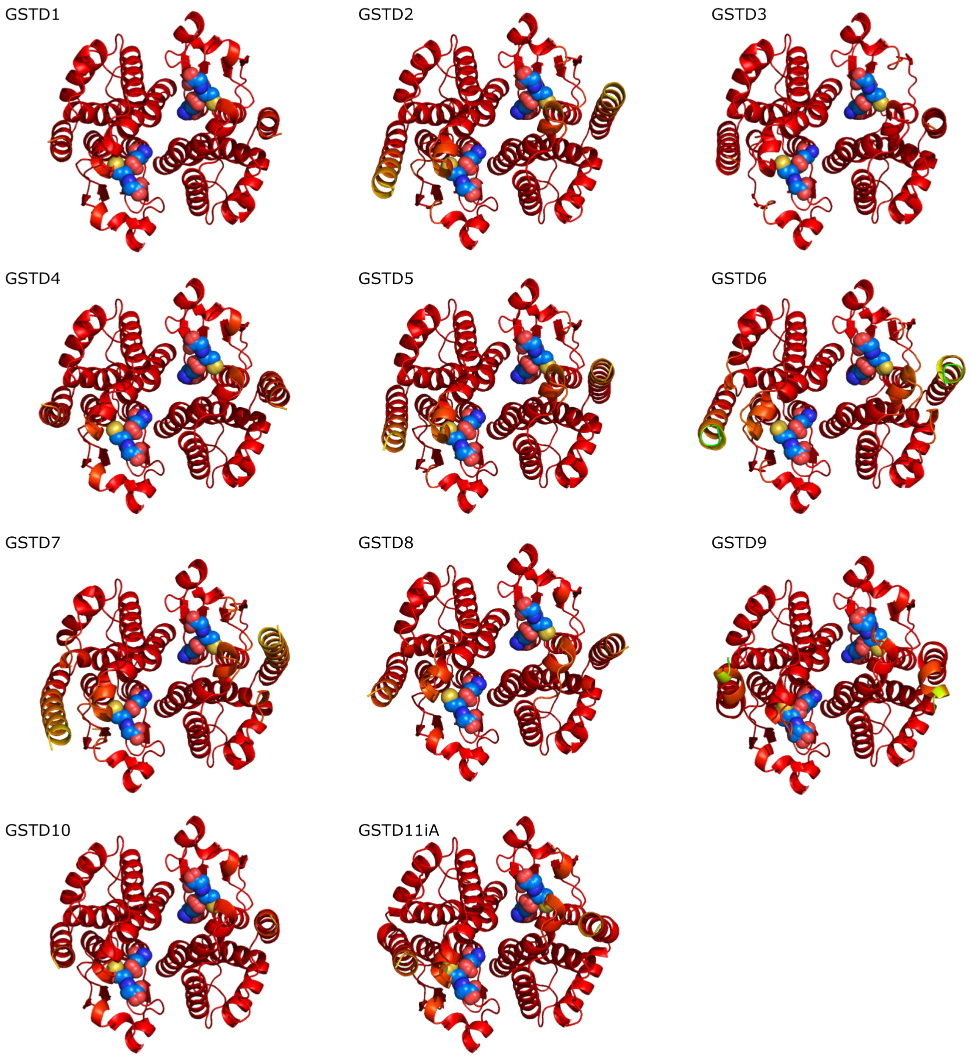

Figure S4. Cartoon representation of GST structures of class δ from *D. mel* and predicted by AlphaFold.

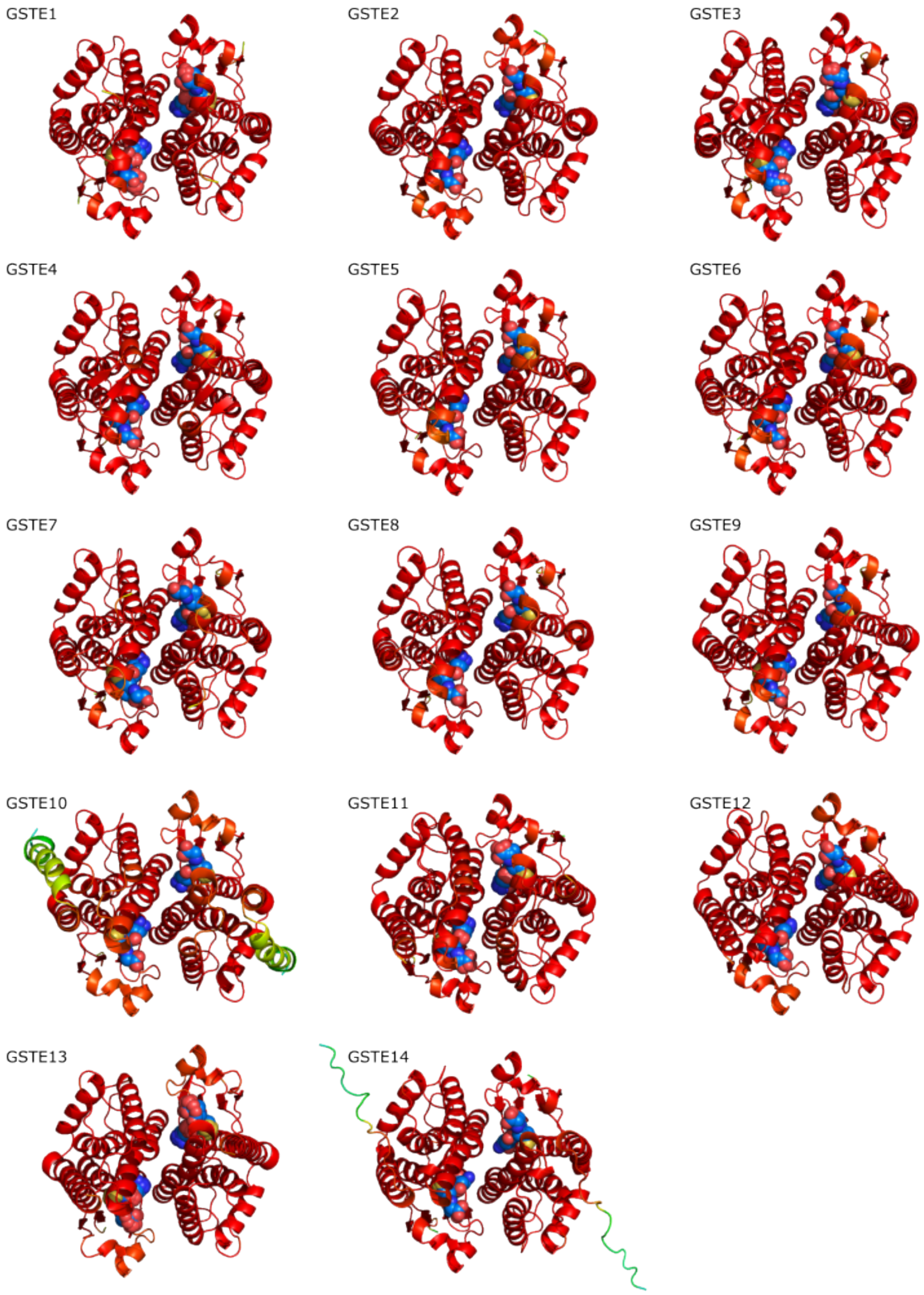

Figure S5. Cartoon representation of GST structures of class ε from *D. mel* and predicted by AlphaFold.

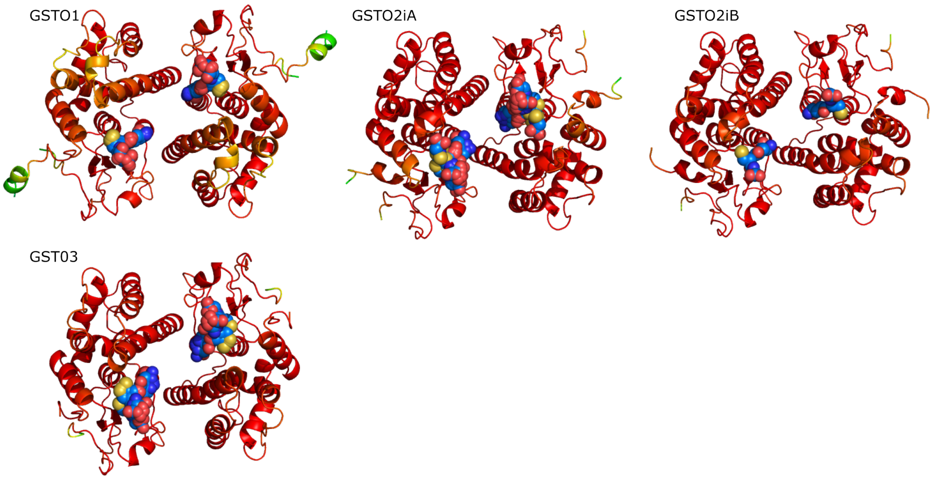

Figure S6. Cartoon representation of GST structures of class ω from *D. mel* and predicted by AlphaFold.

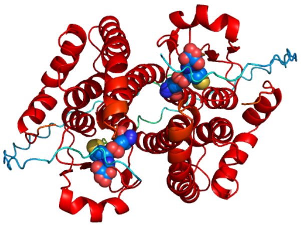

Figure S7. Cartoon representation of GST structures of class σ from *D. mel* and predicted by AlphaFold.

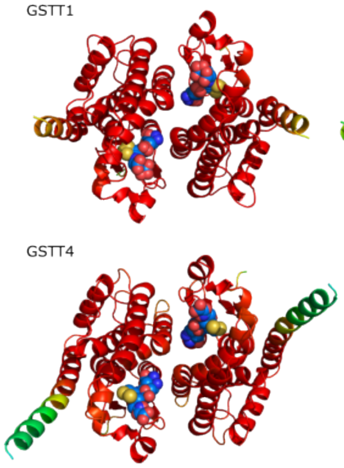

Figure S8. Cartoon representation of GST structures of class θ from *D. mel* and predicted by AlphaFold.

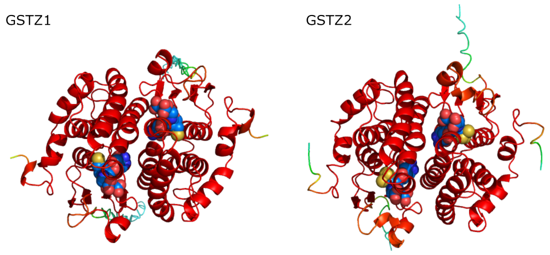

Figure S9. Cartoon representation of GST structures of class ζ from *D. mel* and predicted by AlphaFold.

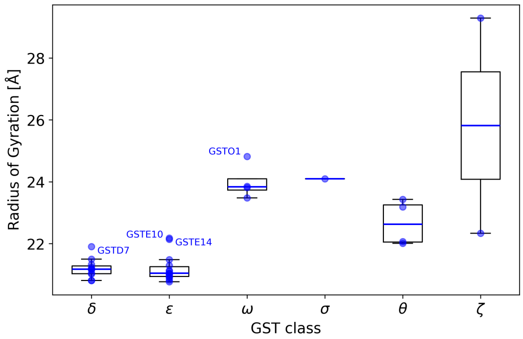

Figure S10. Radius of gyration (in Å) as a function of GST class for the D. mel GSTome

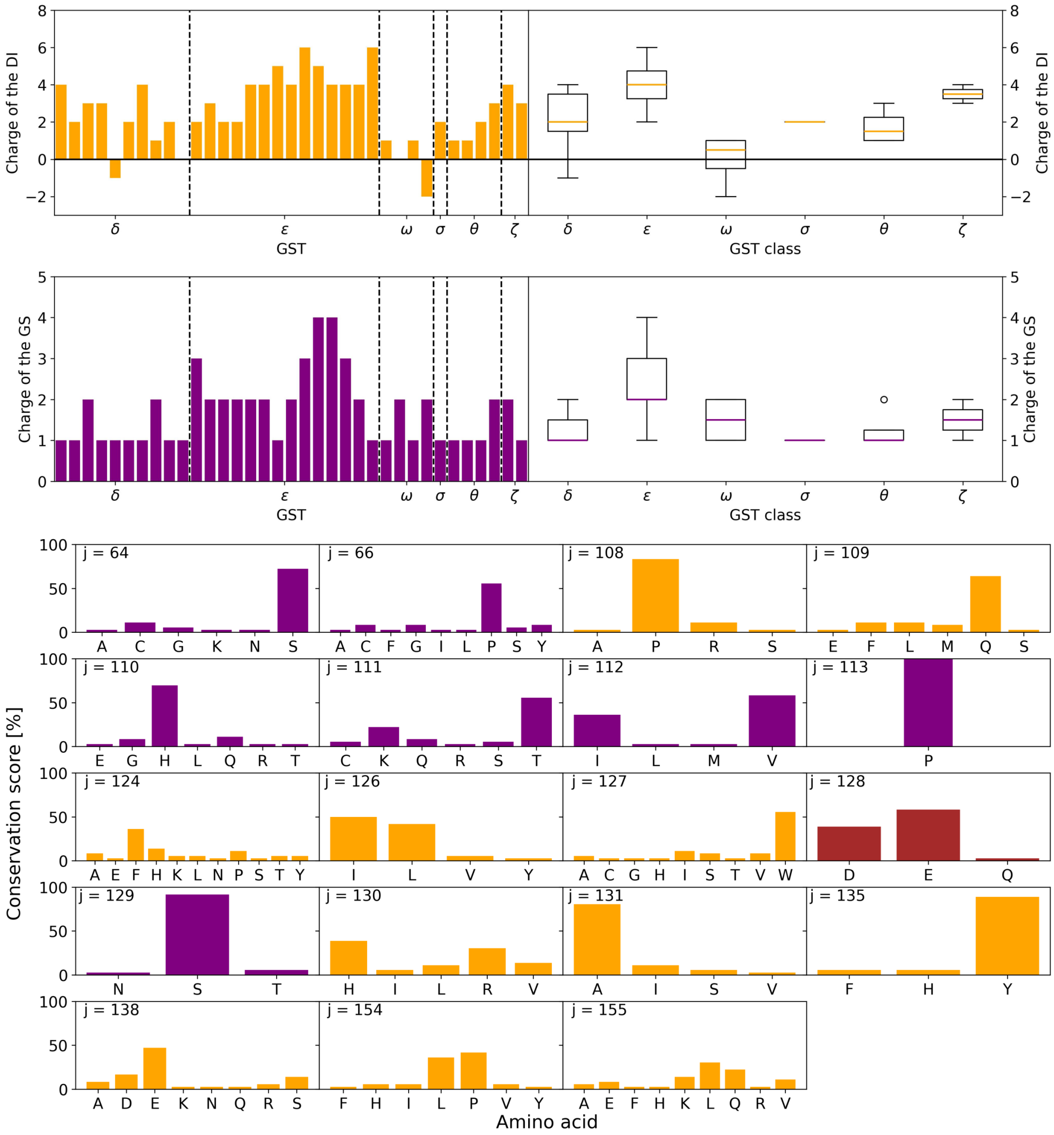

Figure S11. Charge analysis and conservation plot of the residues in Dimerization interface (orange) and in the Glutathione Binding Site (purple).

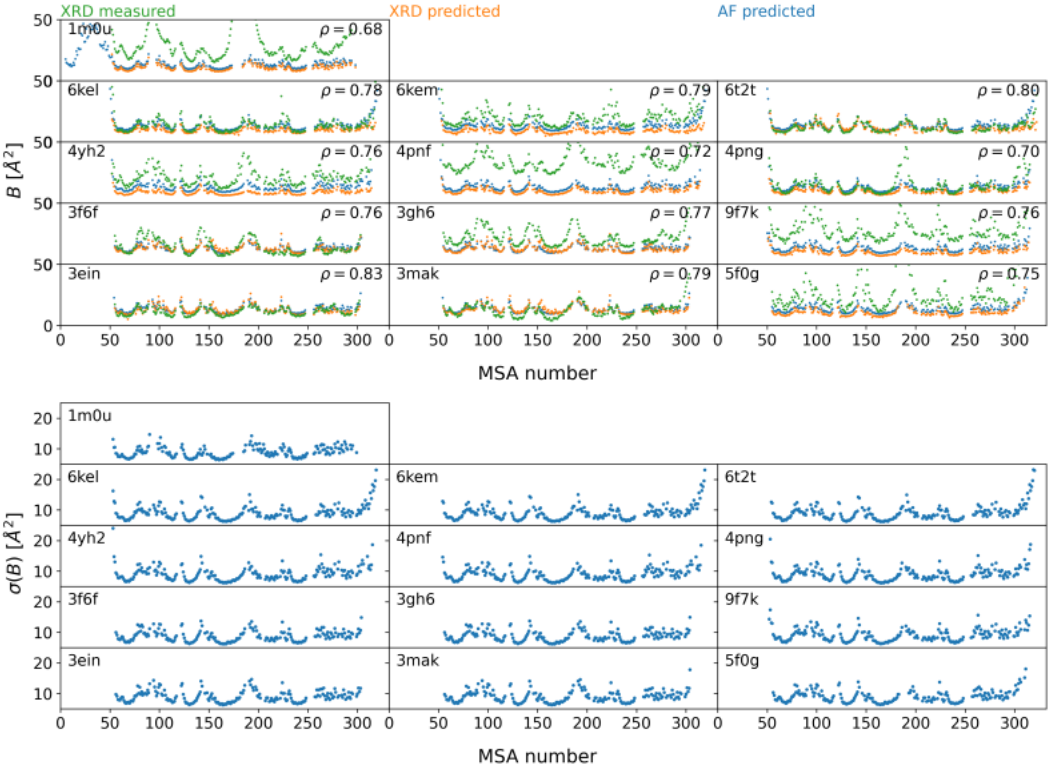

Figure S12. Thermal B-factors (in Å^2) and their standard deviation (in Å^2) as a function of MSA number computed for the 12 GST experimental structures presented in Tab. 1.
